# Efficacy and Durability of Intratympanic Gentamicin Treatment for Meniere's Disease

**DOI:** 10.3389/fneur.2021.765208

**Published:** 2021-12-09

**Authors:** Yafeng Guan, Divya A. Chari, Yu-Hsi Liu, Steven D. Rauch

**Affiliations:** ^1^Department of Otolaryngology, The Hong Kong University-Shenzhen Hospital, Shenzhen, China; ^2^Department of Otolaryngology – Head and Neck Surgery, Massachusetts Eye and Ear, Boston, MA, United States; ^3^Department of Otolaryngology, UMass Memorial Medical Center, Worcester, MA, United States; ^4^Department of Otolaryngology, University of Massachusetts Medical School, Worcester, MA, United States; ^5^Department of Otolaryngology – Head & Neck Surgery, Harvard Medical School, Boston, MA, United States; ^6^Department of Otolaryngology – Head and Neck Surgery, Kaohsiung Vetereans General Hospital, Kaohsiung City, Taiwan

**Keywords:** gentamicin, Meniere's disease, intratympanic (IT) injection, intratympanic treatment, head thrust test

## Abstract

**Objective:** To study the success of intratympanic gentamicin (ITG) treatment in reducing vertigo attacks in Meniere's disease (MD) and the value of the Halmagyi head thrust test (HTT) in predicting treatment durability.

**Study Design:** Retrospective cohort study.

**Setting:** Tertiary care vestibular clinic.

**Patients:** Unilateral MD patients treated with ITG from 2006–2019 with ≥6 months follow-up.

**Main Outcome Measures:** Demographics, audiometric data, subjective symptomatology, and HTT results were collected. Treatment success was defined as sufficient symptom relief. Treatment failure indicated vertigo control of less than 6 months duration. Treatment relapse indicated vertigo recurrence after 6 months.

**Results:** Of 255 patients, treatment success, failure, and relapse occurred in 226 (88.6%), 29 (11.4%), and 121 (47.1%) patients, respectively. 48 (18.8%) patients who failed to respond or relapsed underwent labyrinthectomy. Mean follow-up time was 3.7 yrs (range 0.5–12.8). After ITG treatment, 25% patients reported worse hearing; mean pure tone average (PTA) increased by 18.6 ± 11.3 dB and mean word recognition score (WRS) decreased by 33 ± 21%. Of the 148 patients with negative pre-treatment HHT, 103 (69.6%) converted to positive after ITG treatment. Mean time-to-relapse in the converted and non-converted HTT cohorts was significantly different (49.7 vs. 27.0 months, *p* = 0.009) even after adjusting for gender, age, laterality, duration of symptoms, and number of ITG treatments. There were no significant differences between the two groups in hearing outcomes or subjective symptoms (e.g. lingering disequilibrium).

**Conclusions:** ITG treatment effectively reduces the number of vertigo attacks in MD. HTT is valuable in predicting durability of treatment benefit.

## Introduction

Meniere's disease (MD) causes fluctuating and progressive sensorineural hearing loss (SNHL) and episodic vertigo and can be clinically challenging to manage. For the vast majority of patients with active MD symptoms, it is the episodic vertigo that is most disabling and has the most profound impact on quality of life. Fortunately, treatment of MD is most effective in the control of these acute vertigo attacks, while auditory symptoms such as aural fullness, tinnitus, and hearing loss tend to be less responsive to treatment interventions (1).

In our center, we adopt a conservative approach to diagnostic testing and treatment intervention, and embrace a patient-centered outcome philosophy (2). Our initial treatment intervention for MD patients with intractable vertigo is to provide guidance about trigger management. The three pillars of trigger management are (i) adoption of a regular daily schedule to get the body in a rhythm day-to-day, (ii) redress of any other medical or psychological health issues, and (iii) dietary recommendations to help maintain a constant fluid and electrolyte status to avoid overtaxing the homeostatic systems of their fragile MD ear (3, 4). Patients who continue to suffer vertigo attacks despite undergoing these conservative lifestyle modifications are prescribed a diuretic (5). In our experience, the combination of trigger management and diuretic leads to adequate control of symptoms in the majority of patients. Worldwide, betahistine is widely used as maintenance therapy to reduce or prevent MD attacks. However, it is not approved for use by the U.S Food and Drug Administration because of the quality of evidence for its efficacy in MD is poor (1). Thus, it is problematic for us to prescribe it and for our patients to obtain it. We do not use it. In this remaining cohort who fail to control vertigo with diet/lifestyle interventions and diuretic, intratympanic gentamicin (ITG) is our treatment of choice. Intratympanic corticosteroid (ITC) injection may be offered as a preliminary strategy prior to ITG to patients in whom the MD affects their only hearing ear or patients with serviceable hearing in the affected ear (6). There have been numerous reports in the literature of the efficacy of ITG (7). In the small percentage of MD patients who fail to respond to ITG, surgical interventions such as transmastoid labyrinthectomy, endolymphatic sac decompression, and vestibular neurectomy may be offered (8–10).

If treatment intervention is undertaken and the patient returns for follow-up reporting that the symptom control is adequate, we consider this a treatment success. If the patient reports inadequate symptom control and requests additional therapy, we consider this a treatment failure and offer alternative interventions. Although all MD patients new to our clinic are encouraged to have a baseline comprehensive vestibular test battery, which serves as a reference point should they fail to respond to treatment, (3) many patients decline to undergo these diagnostic procedures because of availability, cost, time considerations, or discomfort. We strongly recommend, and most patients accept, some vestibular function testing (in particular, cervical vestibular evoked myogenic potential [cVEMP]) prior to invasive treatment such as ITG or surgery (4, 5). This pre-treatment testing seeks to specifically address two questions: (i) How “sick” is the affected ear, and (ii) is there any indication of occult dysfunction in the unaffected ear. We have shown previously that 25–30% of asymptomatic ears in unilateral MD patients have abnormal cVEMP (11) and have subsequently shown that these asymptomatic ears with abnormal cVEMP have a significantly greater risk of eventual development of active MD (12).

MD follows a fluctuating course and thus it can be challenging to determine whether a reduction in vertigo attacks arises from drug administration or whether it is simply a consequence of the natural history of the disease. We monitor ITG patients for two indicators of drug effect, a subjective indicator of new onset disequilibrium and an objective indicator of new onset of refixation saccades with a Halmagyi head thrust test. Following administration of ITG, patients often notice a “tipsy disequilibrium” that begins approximately 3–5 days after treatment. Specifically, this is new onset of a generalized disequilibrium that is intensified by movement. The sensation is distinctly different from a full-blown Meniere vertigo attack but very similar or identical to the sensation patients experience following a Meniere attack, when they are no longer having vertigo but are still suffering labyrinthine upset that leads to disturbance of their vestibulo-ocular reflexes and general balance. It is presumably due to onset and progression of post-injection deafferentation. The sensation intensifies to peak around 10–14 days and then gradually fades over an additional four weeks. Post-treatment vestibular function testing may be used to determine the drug effect, but this process can be costly and time-consuming and vestibular function testing is not available in all clinics. As an alternative, the Halmagyi head thrust test (HTT) may be used in the pre- and post-treatment office assessment to determine whether the patient's vestibular function has been impacted by the gentamicin. ITG is typically administered with the goal of hastening reduction of vestibular function within the inner ear. Somewhat analogous to the Fast Forward button on a video player, ITG accomplishes in a matter of weeks the “burn out” that would normally take many years. Conversion of a negative (normal) pre-treatment HTT to a positive (abnormal) post-treatment result indicates some definite degree of peripheral vestibular hypofunction induced by the drug. The HTT is a facile test that can be performed in the clinical setting without specialized equipment.

Herein, we retrospectively review the short- and long-term audiologic and vestibular outcomes following ITG treatment in individuals with unilateral MD and investigate the value of the HTT in predicting durability of ITG treatment benefit.

## Methods

### Study Design

A retrospective chart review was conducted for all MD patients treated with ITG from 2006–2019. Inclusion criteria included patients with unilateral MD as defined by previously published practice guidelines (1) with at least 6 months follow-up after administration of ITG. Pre- and post-treatment audiometric data, subjective vestibular symptomatology such as vertigo, dizziness, and/ or disequilibrium within one month of treatment, and HTT results were recorded along with demographic information. Pre-treatment audiograms were obtained within 1–2 weeks prior to ITG administration. Subjects who complained of subjective hearing loss underwent a post-treatment audiogram, typically around four weeks after treatment. The protocol was deemed exempt by the Massachusetts General Brigham Institutional Review Board (protocol number 2019P003735).

### Intratympanic Gentamicin (ITG) Administration

Patients were positioned supine with the head turned slightly toward the contralateral side. The tympanic membrane was anesthetized at two positions (anterior and posterior) with topical phenol. A ventilation opening is created at the anterior site and 1 mL of room temperature 40 mg/mL gentamicin sulfate was instilled into the posterior site with a 25-gauge spinal needle. The entire 1 mL was instilled, which served to flush air bubbles out through the anterior ventilation opening and fill the middle ear with drug. Excess gentamicin solution that exceeded the volume of the middle ear filled the external auditory canal. Patients remained supine with the treated ear slightly up for one hour and were discharged with instructions to keep the ear dry.

### Definitions of Treatment Effect

**Figure 1:**
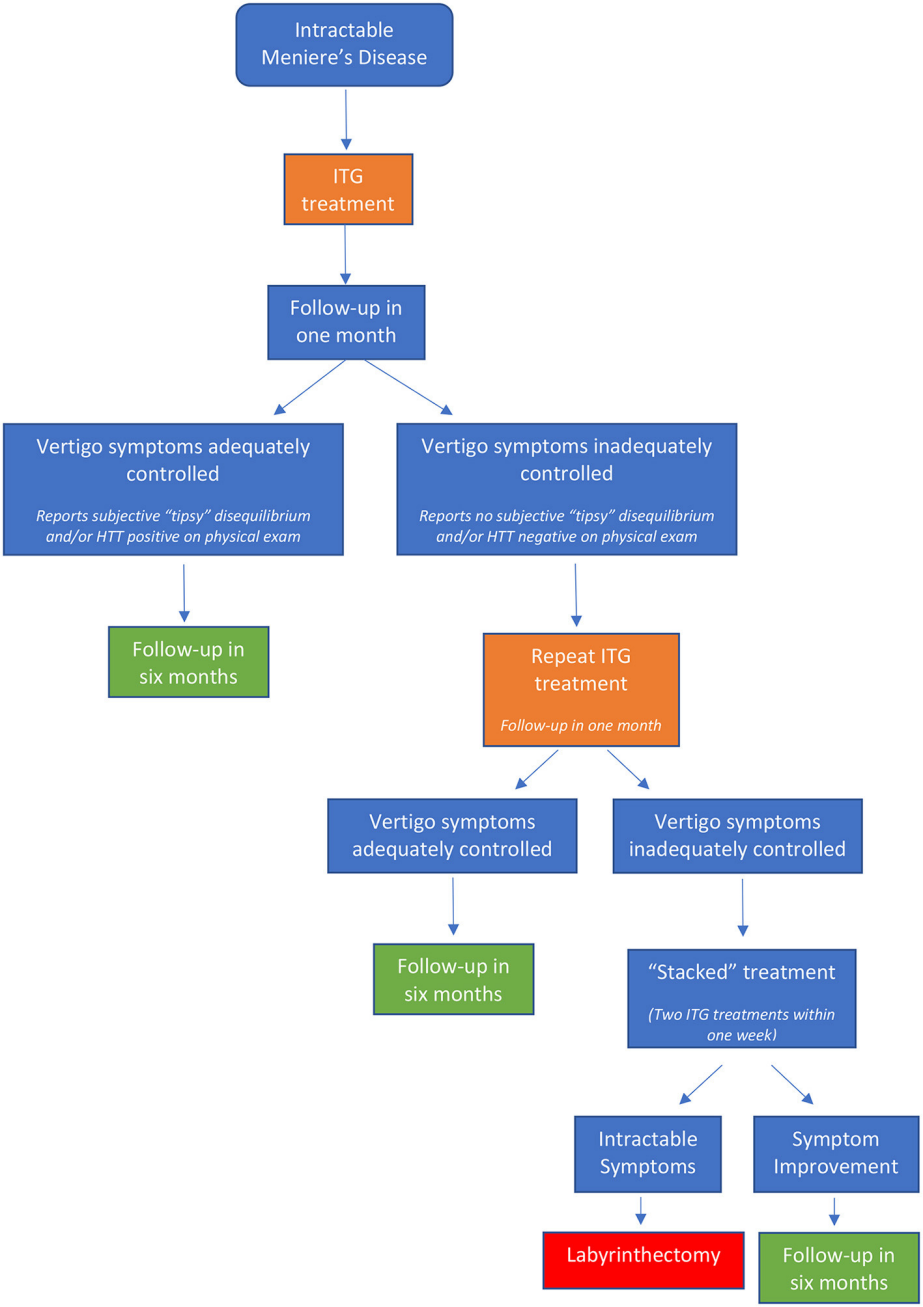
ITG flow chart.

Following ITG administration, patients were categorized into three treatment groups: *success, failure*, and *relapse*. Treatment success included patients who reported adequate abatement of vertigo attacks for at least 6 months and requested no additional treatment or intervention. Treatment failure included patients with vertigo control of less than 6 months duration who required further intervention. Treatment relapse included patients with vertigo recurrence following a quiescent period of more than 6 months after initial treatment (Figure 1).

### Halmagyi Head Thrust Test (HTT)

At the one-month follow-up visit after ITG treatment, an HTT was performed. After confirming that the patient had no significant neck problems and demonstrated normal ocular motility and side-to-side gaze tracking while sitting upright, the patient was asked to fixate their gaze on the examiner's nose. The patient's head was then rotated suddenly and unpredictably in a short, but high velocity horizontal motion to each side. Presence of a refixation saccade was scored as a positive (abnormal) test result. Patients who had a documented pre-treatment negative HTT that subsequently converted to positive post-treatment were categorized into the “converted HTT” group. Patients who had a documented pre-treatment negative HTT that remained negative post-treatment were categorized into the “non-converted HTT” group. In addition to this objective assessment of treatment effect, patients were queried for presence and time course of subjective symptoms of imbalance that were new since the treatment.

### Statistical Analysis

Standard descriptive statistics were used to describe the study population. Statistical analysis was completed using Statistics Package for Social Science (IBM SPSS version 23; SPSS Inc., Chicago, IL) and GraphPad Prism 8 (San Diego, CA). Statistical significance was set at *p* < 0.05. χ^2^ tests were used for comparative analysis of qualitative variables among groups. A Mann Whitney Wilcoxon test and independent two-sample *t*-test were used to compare differences between the converted and non-converted HTT groups. After adjusting for baseline data, including gender, age, laterality, duration of symptoms, and number of ITG treatments, a logistic regression analysis was used to compare the two groups. Comparison of time-to-relapse of the two groups was performed with a Cox proportional hazards regression model.

## Results

### Demographics

A total of 255 unilateral MD subjects met inclusion criteria, all of whom had been treated with ITG. The mean age was 57 yrs (range: 15–83) and there were 135 (52.9%) male subjects. Demographics are shown in Table 1. The mean follow-up period was 3.7 yrs (range: 0.5–12.8 yrs). There were 125 (49.0%) affected right ears. The mean duration of symptoms as calculated from the onset of symptoms was 7.1 yrs (range: 0.3–45 yrs). The number of ITG administrations varied in the cohort; 85 (33.3%) subjects received one injection, 83 (32.6%) subjects received two injections, and 87 (34.1%) subjects received between 3–10 injections.

**Table 1:** Demographics of patients treated with ITG.

**Variable**	**Category**	**Number (%)**	**Total**
Gender	Male Female	135 (52.9%) 120 (47.1%)	255
Age (yrs)	Min Max Mean±SD	15 83 57.4 ± 11.0	255
Laterality	Left Right	130 (51.0%) 125 (49.0%)	255
Length of time with MD (yrs)	Min Max Mean±SD	0.3 45.0 7.1 ± 7.3	244
Number of treatments	Once Twice ≥Three times	85 (33.3%) 83 (32.6%) 87 (34.1%)	255
Drop attack before ITG	Yes No	81 (31.8%) 174 (68.2%)	255
Head thrust test before ITG	Negative Positive	172 (81.5%) 39 (18.5%)	211
Interval until reaction	No reaction Days Weeks	22 (9.8%) 113 (50.4%) 89 (39.7%)	224
Hearing loss level before ITG	None Mild Moderate Severe Profound	3 (1.2%) 26 (10.7%) 109 (44.7%) 103 (42.2%) 3 (1.2%)	244
Hearing loss level after ITG	No Mild Moderate Severe Profound	5 (3.5%) 11 (7.6%) 62 (43.0%) 63 (43.8%) 3 (2.1%)	144
Hearing deterioration after ITG	Yes No	47 (25.0%) 141 (75.0%)	188
Relapse	Yes No	121 (53.5%) 105 (46.5%)	226
Time-to-relapse(months)	6–12 12–24 >24	38 (31.4%) 38 (31.4%) 45 (37.2%)	121
Drop attack after ITG	Yes No	46 (18%) 209 (82.0%)	255
Head thrust test after ITG	Negative Positive	52 (22.6%) 178 (77.4%)	230
Labyrinthectomy after ITG		48 (18.8%)	255
Follow-up time(years)	Min Max Mean ± SD	0.5 12.8 3.7 ± 2.9	255
Lingering disequilibrium	Yes No	26 (10.2%) 229 (89.8%)	255

### Audiometric Outcomes

Prior to ITG treatment, mean pure tone average (PTA; average threshold at 0.5, 1, 2 and 3 kHz) was 57.0 ± 13.7 dB HL and mean word recognition score (WRS) was 49 ± 27 %. 118 patients had audiograms performed after the injection. Of this cohort, 47 (25%) had an objective decline in the performance on audiogram. Of these patients, 11 (23.4%) had one injection, 12 (25.5%) had two injections, and 24 (51.1%) underwent three or more. In these patients, mean PTA increased by 18.6 ± 11.3 dB HL, and mean WRS decreased by 33 ± 21 %.

### Vestibular Outcomes

Before ITG treatment, 81 (31.8%) subjects had experienced at least one drop attack. It is noteworthy that only 11% of all MD patients in the senior author's practice fail diet/lifestyle and diuretic therapy, thereby qualifying for ITG treatment. Thus, a drop attack prevalence of 31.8% is really 31.8% of 11%, or approximately 3.5% of all MD patients. After treatment, 46 (18.0%) patients experienced vertigo and/ or drop attack(s).

We have an elaborate custom cVEMP testing system developed in our Audiology Department over the last 20 years. We test cVEMP threshold for 500, 750, and 1,000 Hz tonebursts. We report these thresholds as “normal” if they fall within the 95% confidence interval for our normative data set, and “abnormal” if they exceed the 95% confidence interval at more than one of the three test frequencies. Of the 189 subjects who underwent pre-treatment cVEMP testing, vestibular hypofunction (i.e. abnormally elevated threshold) was confirmed in 162 (85.7%) affected ears. A total of 255 patients had documented subjective history of their symptoms in the one month following ITG treatment. Of the 202 patients who experienced a “tipsy disquilibrium” reaction, 181 (89.6%) had a successful treatment response of >6 months of adequate vertigo control, and 21 (10.4%) failed. Onset of this reaction varied from days (50.4%) to weeks (39.7%).

Of the 255 patients included in this study, 226 (88.6%) were deemed to be treatment successes (e.g. >6 months of vertigo control) and did not relapse within the observation time window of this study, while 29 (11.4%) were considered treatment failures, and 121 (47.4%) relapsed after an initially successful response. Among the 226 successfully treated patients, 141 (62.4%) required only one injection to achieve vertigo control, 63 (27.9%) required two injections, and 22 (9.7%) required between 3–7 injections (two patients required 5 injections and one patient required 7 injections). Of the 121 patients who eventually relapsed, 38 (31.4%) relapsed in one year, 38 (31.4%) relapsed between one and two years, and 45 (37.2%) relapsed after two years. Among relapsed patients, 100 patients (82.6%) elected to repeat the ITG treatment, with 59 patients achieving successful treatment (e.g. ≥6 months vertigo control), 16 patients failing retreatment, and 25 patients without adequate follow-up time.

Of our 255 patients, 48 patients (18.8%) ultimately underwent surgical transmastoid labyrinthectomy for control of their vertigo attacks after relapse and/or failure of ITG treatments. Twenty-six (10.2%) patients complained of persistent disequilibrium or oscillopsia requiring vestibular physical therapy or chronic and regular use of benzodiazepine central vestibular suppressants.

### HTT Outcomes

#### Baseline Data Analysis

A total of 148 subjects had documented pre-treatment negative HTT. Following ITG treatment, 103 subjects (69.6%) converted to positive HTT (i.e. converted HTT), indicating vestibular hypofunction, while 45 subjects (30.4%) remained negative (i.e. non-converted HTT). Of the 148 subjects, 83 (56.1%) were males with a mean age of 57.3 ± 10.4 yrs old. The mean duration of symptoms is 6.8 ± 7.1 yrs. Disease laterality occurred slightly more frequently on the right side (52 vs. 48%). The mean age of the converted HTT and non-converted HTT groups were 59.0 ± 9.9 yrs and 53.5 ± 10.8 yrs, respectively. See Table 2 for details.

**Table 2:** Baseline characteristics and head thrust response (%) of patients with negative HTT pretreatment.

**Variable**		**Total (%)**	**Post-ITG HTT (+) *n* = 103 (%)**	**Post-ITG HTT (−) *n* = 45 (%)**	**Chi-Square**	***p-value***
Gender	Male	83 (56.1)	57 (55.3)	26 (57.8)	*χ^2^* = 0.076	0.783
	Female	65 (43.9)	46 (44.7)	19 (44.7)		
Side	Left	71 (48.0)	55 (53.4)	16 (35.6)	*χ^2^* = 3.995	0.046
	Right	77 (52.0)	48 (46.6)	29 (64.4)		
Number of Treatments	Once	103 (69.6)	74 (71.8)	29 (64.4)	*χ^2^* = 0.810	0.368
	> Once	45 (30.4)	29 (28.2)	16 (35.6)		
Age (yrs ± SD)		57.32 ± 10.46	58.98 ± 9.92	53.53 ± 10.78	t = 2.993	0.003
Duration of symptoms (yrs ± SD)		6.76 ± 7.09	6.87 ± 7.08	6.50 ± 7.18	z = −0.330	0.741

#### Clinical Treatment Data Analysis

After adjusting for baseline data, including gender, age, laterality, and number of treatments, a logistic regression was used to compare the converted and non-converted HTT groups. Notably, patients who reported experiencing a “tipsy disequilibrium” following drug administration were more likely to convert to positive HTT (*beta* = 1.851, OR 6.363 [95% CI: 1.615–25.080], *p* = 0.008).

There were no significant differences between the groups in drop attacks or conventional MD vertigo attacks (*p* > 0.05). See Table 3 for details.

**Table 3:** Comparison of treatment response in patients who did and did not convert to head thrust (+) after ITG. (%)^*^.

**Variable**		**Post-ITG Head Thrust**		***B***	***P***	***Exp(B) 95% CI***
		**(+)**	**(−)**			
Treatment reaction	Yes	93 (95.9)	29 (78.4)	1.851	0.008	6.363 (1.615,25.080)
	No	4 (4.1)	8 (21.6)			
Drop attack control	Yes	24 (58.5)	8 (47.1)	−0.532	0.483	0.588 (0.133,2.598)
	No	17 (41.5)	9 (52.9)			
Effect	Yes	92 (89.3)	43 (95.6)	−19.171	0.996	—
	No	11 (10.7)	2 (4.4)			

* *Logistic regression analysis was used to adjust baseline data such as gender, age, laterality, duration of symptoms, and number of treatments. Treatment reaction refers to the experience of “tipsy disequilibrium” of post injection deafferentation. Effect refers to whether the symptoms of vertigo were adequately controlled*.

#### Comparison of Time-to-Relapse

**Figure 2:**
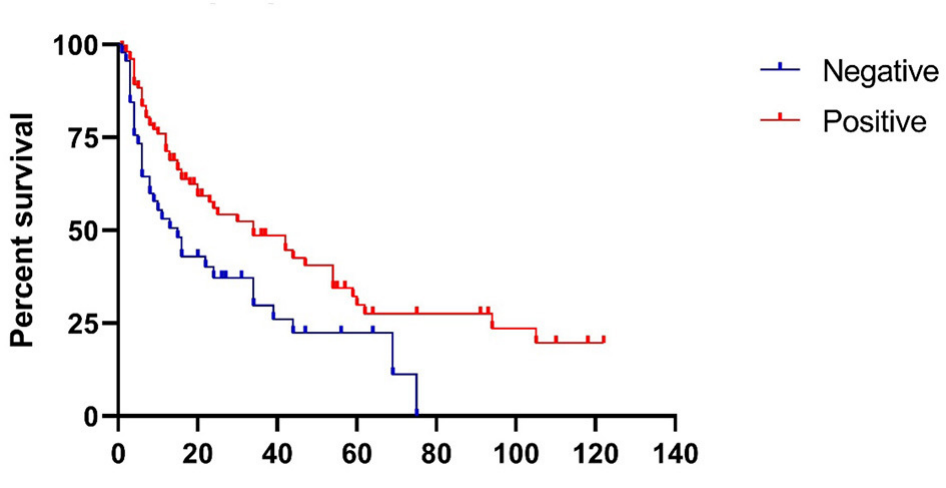
Survival proportions: Survival of ITG treatment patients with different head thrust test results.

Survival curve of time-to-relapse (TTR) in the 148 patients with initial negative pre-treatment HTT and a recorded post-treatment HTT result was used to compare those with a converted HTT vs. non-converted HTT after ITG treatment (see Figure 2). Patients were included in this analysis regardless of whether treatment was successful or not, as long as the pre-treatment HTT was negative and the post-treatment HTT was available. By definition, all patients who “relapsed” in less than 6 months were treatment failures and those relapsing after 6 months were initially scored as treatment success. The survival curves of the two groups were significantly different (*p* = 0.009). The mean TTR of the converted and non-converted HTT groups was 49.7 (95% CI: 39.0–60.5) months vs. 27.0 (95% CI: 18.4–33.6) months, respectively. The median TTR of the converted and non-converted HTT groups was 34 (95% CI: 15.7–52.3) months vs. 15 (95% CI: 7.884–22.116) months, respectively. After adjusting for baseline characteristics, such as gender, age, laterality, duration of symptoms, and number of ITG treatments, the TTR of the converted HTT group was still significantly longer than that of the non-converted group (HR 2.047 [95% CI: 1.277–3.283]).

#### Indicators of Drug Effect

After adjusting for baseline characteristics, logistic regression was used to compare the subjective drug effect of post-treatment disequilibrium reaction and objective drug effect of hearing decline (threshold elevation and/or decreased word recognition score) between the converted vs. non-converted HTT groups. There were no significant differences (*p* > 0.05) between the two groups. See Table 4 for details.

**Table 4:** Comparison of side effects between the positive and negative groups of the post-ITG treatment HTT (%)^*^.

**Variable**		**Positive HTT**	**Negative HTT**	***B***	***p-value***	***Exp(B)* 95% *CI***
Post-ITG Disequilibrium	Yes	8 (7.8)	5 (11.1)	−0.658	0.317	1.930 (0.532–6.996)
	No	95 (92.2)	40(88.9)			
PTA Worse	Yes	29 (42.0)	14 (60.9)	−0.108	0.853	0.898 (0.285–2.826)
	No	21 (58.0)	9(39.1)			
WRS Decrease	Yes	31 (62.0)	14 (60.9)	0.229	0.698	1.257 (0.395–4.001)
	No	19 (38.0)	9 (39.1)			

* *Logistic regression analysis was used to adjust baseline data such as gender, age, side, duration of symptoms, and number of treatments*.

## Discussion

This study had two primary aims: (1) to characterize the audiologic and vestibular outcomes after ITG treatment in individuals with unilateral MD and (2) to study the value of HTT in predicting durability of ITG treatment benefit. Following drug administration, treatment success, failure, and relapse occurred in 41.2%, 11.4%, and 47.4% of patients, respectively. After ITG treatment, about one-quarter of patients developed worsened hearing. Of the patients who had documented pre-treatment negative HTT, 69.6% converted to positive HTT after treatment. Notably, the mean time-to-relapse in the converted HTT group was significantly greater than that of the non-converted HTT group. These results suggest that an abnormal post-treatment result on HTT may predict a longer-lasting treatment effect. This finding did not change even after statistical adjustments were made to account for differences in age and the number of ITG treatments. The converted and non-converted HTT groups did not differ significantly in other audiometric or vestibular (number of vertigo episodes or drop attacks) metrics nor on subjective symptoms such as persistent disequilibrium.

Although Schuknecht et al. reported use of intratympanic aminoglycosides for MD in the 1950s (13), this form of therapy did not gain widespread popularity until the past several decades. Gentamicin is an aminoglycoside antibiotic that has both vestibulotoxic and cochleotoxic effects, specifically targeting the sensory cells of the vestibular apparatus and the hair cells in the cochlea, with a stronger predilection for the vestibular system (14). Current clinical practice guidelines suggest that intratympanic gentamicin be offered to patients with MD who do not respond to more conservative measures, such as trigger management and diuretics (1). There have been two double-blinded randomized control trials investigating the effect of ITG on vertigo control (15, 16). Both studies reported a significant reduction in vertigo complaints, with an average increase in hearing loss of 18.1 dB HL following ITG. As in prior studies, we found a significant reduction in vertigo symptoms following ITG, with a mean threshold shift of +18.6 dB HL in the 25% of ITG-treated ears who suffered hearing loss.

The head thrust test (HTT) is a widely accepted clinical test that may be used to assess the angular vestibulo-ocular reflex (VOR). In this study, the HTT was used to evaluate the function of the horizontal semicircular canal, as described by Halmagyi and Curthoys in 1998 (17). In individuals with normal horizontal semicircular canal function, abrupt rotation of the head toward the unaffected side has no effect on gaze targeting. However, when there is hypofunction of the horizontal semicircular canal, abrupt rotation toward the affected side will cause the gaze to deviate off target in the same direction as the head movement followed by a corrective saccade to re-establish gaze fixation. For patients with complete unilateral vestibular hypofunction, the sensitivity and specificity of HTT is 100% (18). However, in patients with non-surgically-induced unilateral vestibular hypofunction, such as may occur in cases of vestibular neuritis or following administration of vestibulotoxic medications (e.g. gentamicin), the sensitivity and specificity is estimated to be around 34–39% and 95–100%, respectively (19, 20).

Prior studies have attempted to determine whether vestibular function testing can predict the outcome of ITG treatment. Indeed, greater vestibular dysfunction has been found to correlate with better long-term vertigo control (21, 22). In a study of 20 patients with unilateral MD, Martin-Sanz et al. sought to determine whether changes in the VOR gain after ITG could be correlated with vertigo control (23). The authors found a significant correlation between the 1-month post-treatment VOR gain and the rate of vertigo recurrence after the first ITG treatment. A prospective cohort study of 25 patients with MD with VEMP and caloric testing pre- and post-treatment with ITG showed that absent VEMPs and caloric responses after treatment were correlated with significant symptom improvement at 6-month follow up (24).

Although routine use of formal vestibular function testing for patients with MD is not required, these tests may provide complementary information to help lateralize MD and assess the vestibular system prior to, during, and after ablative procedures. However, not all clinics have the access nor resources to provide vestibular function testing for patients and moreover, patients may refuse to undergo testing due to cost, time considerations, or discomfort. By contrast, the HTT is a simple clinical test that requires no specialized equipment. In our study, we found that the development of a positive (abnormal) HTT response after ITG was correlated with a significantly longer median time-to-relapse (34 vs. 15 months).

The mechanism of relapse after ITG treatment or “gentamicin resistance” is not known. Some authors have suggested that variability in the permeability of the round window or the presence of a pseudo-membrane over the round window may account for cases of relapse or failure (25, 26). Several animal studies have demonstrated that vestibular hair cells may regenerate after injury or damage, but it is unknown whether this regenerative process would lead to functional recovery in humans. Successful ITG treatment causes significant reduction of peripheral vestibular function in the treated ear and effectively mitigates Meniere vertigo attacks in the majority of cases. Recovery from the acute effects of this peripheral vestibular hypofunction depend upon central vestibular compensation, which in turn depends in part upon integrity of vestibular function in the contralateral ear. Since no less than 25–30% of patients with unilateral MD are expected to eventually develop involvement of the second ear, it is possible that the asymptomatic ear may already be slightly affected. If so, ITG treatment could result in bilateral hypofunction, a much more debilitating condition. Pre-treatment vestibular testing can help spot such cases and at least with cVEMP, can offer some predictive information (but not certainty) about future involvement of the second ear. Informed consent discussion with each patient includes this information. However, it is the disabling vertigo or drop attacks from the currently active ear that impacts each patient's quality of life and justifies ITG treatment. Rarely do considerations of hypothetical future problems alter treatment recommendations for current and actual symptoms.

Limitations of this study include the retrospective study design, the subjectivity of the HTT test, and the lack of complete pre- and post-treatment vestibular function testing in this patient population. Of note, all HTT tests were performed by a single, senior physician with expertise in vestibular disorders. There may have been some inter-subject variability as quantitative data was not obtained from the HTT. A formal video head impulse test (vHIT) (25), which is a well-validated clinical measure of the vestibulo-ocular reflex, was not performed. However, the vHIT machine is not easily accessible in all clinics, whereas the HTT, which can be performed in the clinical setting without specialized equipment, has tremendous utility, particularly in resource-limited settings.

ITG treatment is effective in reducing the number of vertigo episodes and drop attacks in MD. HTT is a valuable clinical tool that is predictive of the durability of the ITG treatment benefit.

## Data Availability Statement

The raw data supporting the conclusions of this article will be made available by the authors, without undue reservation.

## Ethics Statement

The studies involving human participants were reviewed and approved by Partners Healthcare IRB Protocol# 2019P003735. Written informed consent for participation was not required for this study in accordance with the national legislation and the institutional requirements.

## Author Contributions

YG, DC, Y-HL, and SR contributed to study design, data analysis and interpretation, and writing of the manuscript. YG collected all the study data and created the figures and data tables. All authors contributed to the article and approved the submitted version.

## Funding

This research was supported in part by a grant from China Scholarship Council (YG) and by the senior author's (SR) discretionary research funds.

## Conflict of Interest

The authors declare that the research was conducted in the absence of any commercial or financial relationships that could be construed as a potential conflict of interest.

## Publisher's Note

All claims expressed in this article are solely those of the authors and do not necessarily represent those of their affiliated organizations, or those of the publisher, the editors and the reviewers. Any product that may be evaluated in this article, or claim that may be made by its manufacturer, is not guaranteed or endorsed by the publisher.

## References

[1] Basura GJ Adams ME Monfared A Schwartz SR Antonelli PJ Burkard R. Clinical practice guideline: meniere's disease executive summary. Otolaryngol Head Neck Surg. (2020) 162:415–34. 10.1177/0194599820909439 32267820

[2] Minor LB Schessel DA Carey JP. Meniere's disease. Curr Opin Neurol. (2004) 17:9–16. 10.1097/00019052-200402000-00004 15090872

[3] Espinosa-Sanchez JM Lopez-Escamez JA. Meniere's disease. Handb Clin Neurol. (2016) 137:257–77. 10.1016/B978-0-444-63437-5.00019-4 27638077

[4] Lopez-Escamez JA Batuecas-Caletrio A Bisdorff A. Towards personalized medicine in Meniere's disease. F1000Res. (2018) 7:F1000. 10.12688/f1000research.14417.1 30430003 PMC6097350

[5] Crowson MG Patki A Tucci DL. A systematic review of diuretics in the medical management of Meniere's disease. Otolaryngol Head Neck Surg. (2016) 154:824–34. 10.1177/0194599816630733 26932948

[6] Patel M Agarwal K Arshad Q Hariri M Rea P Seemungal BM. Intratympanic methylprednisolone versus gentamicin in patients with unilateral Meniere's disease: a randomised, double-blind, comparative effectiveness trial. Lancet. (2016) 388:2753–62. 10.1016/S0140-6736(16)31461-1 27865535

[7] Pullens B van Benthem PP. Intratympanic gentamicin for Meniere's disease or syndrome. Cochrane Database Syst Rev. (2011) 3:CD008234. 10.1002/14651858.CD008234.pub2 21412917 PMC13378876

[8] Bergmark RW Semco RS Abdul-Aziz D Rauch SD. Transmastoid Labyrinthectomy for Meniere's Disease: Experience and Outcomes. Otol Neurotol. (2020) 41:1413–8. 10.1097/MAO.0000000000002805 32810022

[9] Pullens B Verschuur HP van Benthem PP. Surgery for Meniere's disease. Cochrane Database Syst Rev. (2013) 2:CD005395. 10.1002/14651858.CD005395.pub3 23450562 PMC7389445

[10] Sood AJ Lambert PR Nguyen SA Meyer TA. Endolymphatic sac surgery for Meniere's disease: a systematic review and meta-analysis. Otol Neurotol. (2014) 35:1033–45. 10.1097/MAO.0000000000000324 24751747

[11] Lin MY Timmer FCA Oriel BS Zhou G Guinan JJ Kujawa SG. Vestibular evoked myogenic potential (VEMP) can detect asymptomatic saccular hydrops. Laryngoscope. (2006) 116:987–92. 10.1097/01.mlg.0000216815.75512.03 16735912 PMC2758415

[12] Noij KS Hermann BS Guinan JJ Rauch SD. Predicting development of bilateral Meniere's disease based on cVEMP thresholds and tuning. Otol Neurotol. (2019) 40:1346–52. 10.1097/MAO.0000000000002375 31568134

[13] Schuknecht HF. Ablation therapy in the management of Meniere's disease. Acta Otolaryngol Suppl. (1957) 132:1–42. 13457879

[14] Salt AN. Pharmacokinetics of drug entry into cochlear fluids. Volta Rev. (2005) 105:277–98. 17330152 PMC1805693

[15] Postema RJ Kingma CM Wit HP Albers FW Van Der Laan BF. Intratympanic gentamicin therapy for control of vertigo in unilateral Menire's disease: a prospective, double-blind, randomized, placebo-controlled trial. Acta Otolaryngol. (2008) 128:876–80. 10.1080/00016480701762458 18607963

[16] Stokroos R Kingma H. Selective vestibular ablation by intratympanic gentamicin in patients with unilateral active Meniere's disease: a prospective, double-blind, placebo-controlled, randomized clinical trial. Acta Otolaryngol. (2004) 124:172–5. 10.1080/00016480410016621 15072419

[17] Halmagyi GM Curthoys IS. A clinical sign of canal paresis. Arch Neurol. (1988) 45:737–9. 10.1001/archneur.1988.00520310043015 3390028

[18] Foster CA Foster BD Spindler J Harris JP. Functional loss of the horizontal doll's eye reflex following unilateral vestibular lesions. Laryngoscope. (1994) 104:473–8. 10.1288/00005537-199404000-00013 8164488

[19] Beynon GJ Jani P Baguley DM. A clinical evaluation of head impulse testing. Clin Otolaryngol Allied Sci. (1998) 23:117–22. 10.1046/j.1365-2273.1998.00112.x 9597280

[20] Harvey SA Wood DJ. The oculocephalic response in the evaluation of the dizzy patient. Laryngoscope. (1996) 106:6–9. 10.1097/00005537-199601000-00002 8544630

[21] De Waele C Meguenni R Freyss G Zamith F Bellalimat N Vidal PP. Intratympanic gentamicin injections for Meniere disease: vestibular hair cell impairment and regeneration. Neurology. (2002) 59:1442–4. 10.1212/WNL.59.9.1442 12427902

[22] Hone SW Nedzelski J Chen J. Does intratympanic gentamicin treatment for Meniere's disease cause complete vestibular ablation? J Otolaryngol. (2000) 29:83–7. 10819105

[23] Martingano D Renson A Rogoff S Singh S Kesavan Nasir M Kim J. Daily gentamicin using ideal body weight demonstrates lower risk of postpartum endometritis and increased chance of successful outcome compared with traditional 8-hour dosing for the treatment of intrapartum chorioamnionitis. J Matern Fetal Neonatal Med. (2019) 32:3204–8. 10.1080/14767058.2018.1460348 29642754

[24] Gode S Celebisoy N Akyuz A Gulec F Karapolat H Bilgen C. Single-shot, low-dose intratympanic gentamicin in Meniere disease: role of vestibular-evoked myogenic potentials and caloric test in the prediction of outcome. Am J Otolaryngol. (2011) 32:412–6. 10.1016/j.amjoto.2010.07.021 20851502

[25] Silverstein H Arruda J Rosenberg SI Deems D Hester TO. Direct round window membrane application of gentamicin in the treatment of Meniere's disease. Otolaryngol Head Neck Surg. (1999) 120:649–55. 10.1053/hn.1999.v120.a91763 10229588

[26] Yoshioka M Naganawa S Sone M Nakata S Teranishi M Nakashima T. Individual differences in the permeability of the round window: evaluating the movement of intratympanic gadolinium into the inner ear. Otol Neurotol. (2009) 30:645–8. 10.1097/MAO.0b013e31819bda66 19415042

